# Using longitudinal survey and sensor data to understand the social and ecological determinants of clean fuels use and discontinuance in rural Ghana

**DOI:** 10.1088/2515-7620/abb831

**Published:** 2020-09-28

**Authors:** D Carrión, R Prah, C F Gould, F Agbokey, M Mujtaba, A Pillarisetti, M Tumasi, O Agyei, S Chillrud, T Tawiah, D Jack, K P Asante

**Affiliations:** 1 Department of Environmental Medicine and Public Health, Icahn School of Medicine at Mount Sinai, New York, United States of America; 2 Kintampo Health Research Centre, Kintampo, Ghana; 3 Department of Environmental Health Sciences, Columbia University, New York, United States of America; 4 Gangarosa Department of Environmental Health, Rollins School of Public Health, Emory University, Atlanta, Georgia, United States of America; 5 Lamont-Doherty Earth Observatory, Columbia University, New York, United States of America

**Keywords:** biomass combustion, sustained use, clean cookstoves, discontinued use, energy access, energy transitions

## Abstract

Efforts to reduce the health and ecological burdens of household biomass combustion are underway in Ghana, principally by promoting clean cookstoves and fuels. Recent studies have focused on the sustained use of clean cookstoves, but sometimes household adopt a new cookstove and then end use of that stove. In this study, we introduce a novel framework for understanding and encouraging household transitions to cleaner cooking: clean fuel discontinuance. We leveraged data from the Ghana Randomized Air Pollution and Health Study (GRAPHS) (N = 1412) where pregnant women received either improved biomass (BioLite) or dual burner LPG stoves for free. LPG users were given free LPG refills during GRAPHS. Weekly questionnaires were administered. Stove use monitors tracked a sub-cohort (n = 220) 6 months before and after the fuel subsidy. We examined social and ecological determinants of stove use and discontinuance. Overall intervention stove use adherence was high throughout GRAPHS, with self-reported use at 69% and 86% of participant-weeks for BioLite and LPG arms respectively. Participants used intervention stoves less for meals requiring vigorous stirring. Burns from intervention stoves decreased use among BioLite (RR: 0.96, p = 0.009), but not LPG users. Device breakage was mentioned as an impediment in 18% of free-text responses for LPG users and 1% for BioLite. Tree canopy within a spatial buffer—a plausible proxy for biomass fuels access—was the only variable explaining LPG discontinued stove use in adjusted Cox time-to-event analyses (HR = −0.56, p < 0.001). Future studies should consider the stove use discontinuance framework.

## 1. Introduction

Increasing the availability and uptake of clean cooking fuels is central to sustainable development (Rosenthal et al 2018). Like many countries in Sub Saharan Africa, Ghana is attempting to decrease household biomass combustion by increasing the use of clean cooking fuels (World Bank 2014, ENERGIA 2015). Ghana established a Sustainable Energy for All policy in 2008, with a target of 50% of the population using liquified petroleum gas (LPG) by 2020 (Energy-Commission 2012). Unfortunately, data suggest that Ghana has been unable to reach those targets (Asante et al 2018). Research is warranted to inform the design of evidence-based policies that result in sustained, exclusive, use of clean stoves and fuels. We utilize a longitudinal cohort from the Ghana Randomized Air Pollution and Health Study (GRAPHS) clean cooking randomized controlled trial (Jack et al 2015) to understand why individuals stop using clean or improved cookstoves even after they already own those stoves.

Initiated in 2013, the Ghana Rural LPG Progamme offers free LPG stoves and cylinders to households in poor rural villages (Asante et al 2018). An evaluation of the program found that less than 5% of beneficiaries used their LPG stoves nine months after delivery (Abdulai et al 2018). While reducing biomass use in rural areas is a worthwhile goal for health and ecological concerns, those benefits can only be realized if replacement stoves are used consistently. Studies demonstrate that near complete displacement of traditional fuels is required to reach World Health Organization guidelines for exposure to particulate matter (Johnson and Chiang 2015).

A body of literature has focused on understanding the determinants of adoption and sustained use of improved and clean cookstoves (Rehfuess et al 2014, Puzzolo et al 2016, Muller and Yan 2018). These studies aim to inform behavioral, programmatic, and policy interventions that increase clean cookstove use. More recently, researchers have proposed a solid-fuel suspension framework to understand determinants of transitioning to exclusive clean fuel use (Carter et al 2020). A gap in this literature is studying which factors cause people to decrease or stop using their clean cookstoves. Several studies have reported that participants stop using clean cookstoves during or soon after a study period (Hanna et al 2016, Tigabu 2017, Mudombi et al 2018), yet formal assessments of the determinants of this clean cookstove abandonment have been minimal (Wang and Corson 2015, Chalise et al 2018). We present an empirical assessment of factors that lead people to decrease and ultimately stop using their clean cookstoves—a phenomenon we term ‘clean cookstove discontinuance’—which we define as disuse of their intervention stove. We utilize this unique study context to inform policies and programs that can prevent or reduce stove discontinuance and maximize the ecological and health gains from the Government of Ghana’s efforts to promote clean cooking.

**Figure 1.**
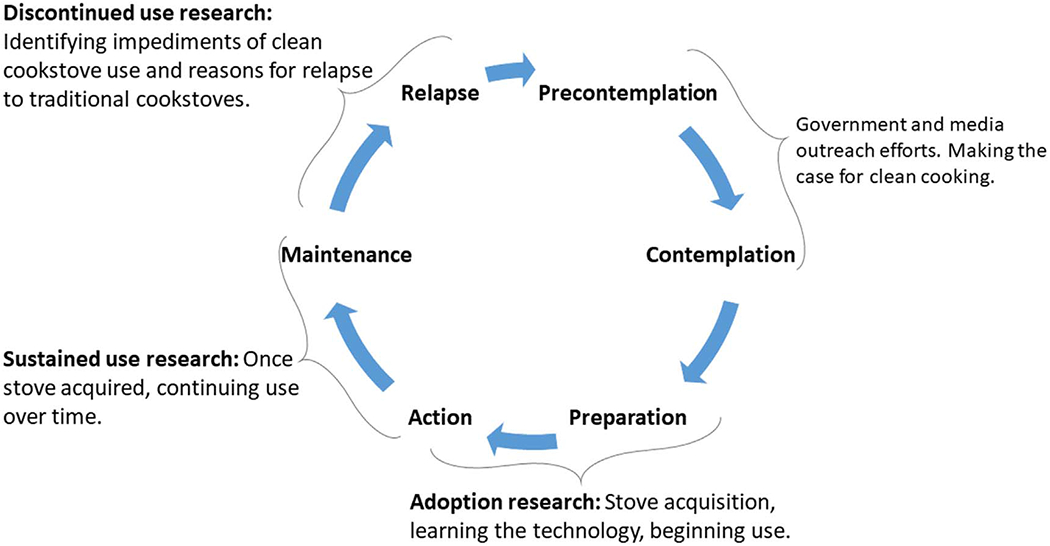
Adapted transtheoretical (Stages of Change) Model for clean cooking.

Stove discontinuance has its foundation in health behavior theory. The Transtheoretical (Stages of Change) Model posits that adoption of healthy behaviors is not linear (Prochaska and Velicer 1997) (figure 1). Instead, behavior change commonly entails relapse to the old behavior. After relapse, some individuals restart efforts towards behavior change while others stop altogether. Studies have investigated the determinants of relapse for physical activity (Marshall and Biddle 2001), changes in diet (Di Noia and Prochaska 2010), smoking cessation (Spencer et al 2002), and water, sanitation, and hygiene (Kraemer and Mosler 2011), but none for clean cookstove use, to the best of our knowledge. Identifying factors that contribute to stove use discontinuance can inform individual, community, and/or national policy interventions to prevent discontinuance or shorten its duration.

We leverage data from GRAPHS to explore barriers to intervention stove use and the social and ecological drivers of improved and clean cookstove discontinuance. Weekly survey data combined with sensor-based stove use monitoring data provide a unique opportunity to explore these constructs in this rural Ghanaian cohort. Our study uses longitudinal data to describe: (1) self-reported stove use patterns, and reasons for disuse, when participants are receiving free LPG fuel; (2) sensor-based stove use patterns before and after the fuel subsidy; and (3) sensor-based measures of clean cookstove discontinuance post-subsidy.

## 2. Methods

### 2.1. Study participants and context

**Figure 2.**
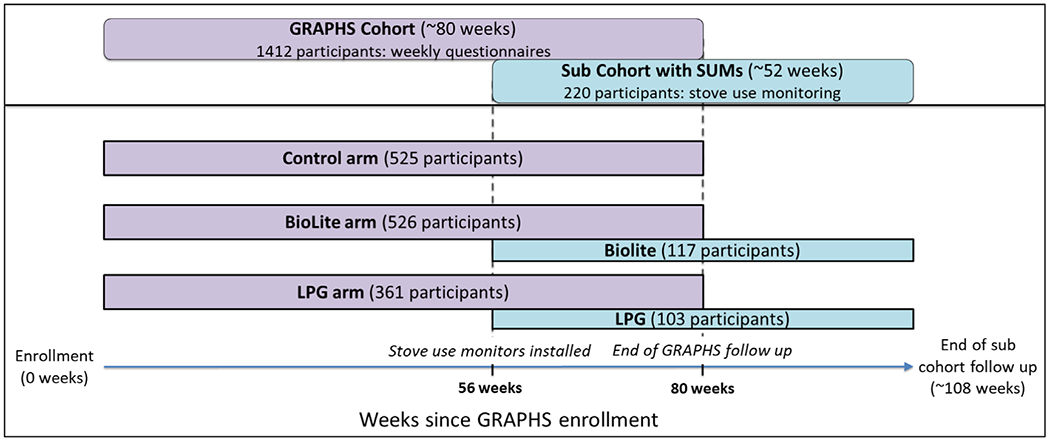
Timeline of GRAPHS and the monitored subcohort, relative to enrollment into GRAPHS. Calendar dates of enrollment were between September 2013 and January 2014.

The participants of this study are a sub-cohort of the Ghana Randomized Air Pollution and Health Study (GRAPHS) (Jack et al 2015). Briefly, GRAPHS was a cookstove intervention trial in rural Kintampo, Ghana that enrolled pregnant women who were cluster-randomized (by community) to receive either two improved biomass stoves (BioLite; Brooklyn, New York), or a dual-burner LPG stove, or to maintain use of their traditional 3-stone fire and/or charcoal stove. A total of 1,414 were enrolled, but two withdrew before baseline data collection, yielding a total sample of 1,412. The BioLite stove is designed to achieve greater efficiency through fan-assisted combustion and the use of smaller-than-typical pieces of biomass fuel. Participants in the LPG arm of the study were provided with free LPG fuel for the duration of the study upon depletion of their LPG cylinder(s). A sub-cohort of 220 participants were randomly chosen from the BioLite (n = 117/526) and LPG (n = 103/361) arms of GRAPHS for the current study (figure 2). These individuals agreed to have stove use monitors (SUMs) installed on their LPG or BioLite stoves for six months prior to the end of their enrollment in GRAPHS and for an additional six months after their participation in GRAPHS ended. Participants in the LPG arm no longer received free LPG fuel after study termination. Therefore, this study offers a unique opportunity to study stove discontinuance in the 6 months after GRAPHS ended when participants had to pay for LPG cylinder refills.

### 2.2. Ethical approvals

Ethical approvals for this study were obtained from the Institutional Review Board of Columbia University Medical Center and the Kintampo Health Research Centre Institutional Ethics Committee.

### 2.3. Self-reported stove use during GRAPHS

#### 2.3.1. Baseline and process data collection

Questionnaires were administered weekly in the participant’s local language by trained field staff throughout the GRAPHS study period. Demographic variables were captured at baseline. For the current analysis, we derived a household asset index as a measure of socioeconomic status (Gunnsteinsson et al 2010). Weekly surveys included stove use questionnaires (supplemental figure 1 is available online at stacks.iop.org/ERC/2/095003/mmedia). Participants were asked if they used the intervention stove (BioLite or LPG) the day preceding the weekly visit. We used these data to summarize intervention stove use throughout the GRAPHS study.

Participants were asked if they had sustained any burns while using the intervention stove. We performed quasi-Poisson regressions, stratified by stove type, to assess weeks of stove use per person and if they had ever experienced a burn. Open-ended questions investigated other reasons for non-intervention stove use. We utilized text analysis to explore these responses (Silge and Robinson 2017). Stop words (commonly used articles and prepositions) were removed during pre-processing. We then generated unique n-grams, specifically bigrams, to offer insight into the sentiment of the short responses (Tan et al 2002). Bigrams are a tool from natural language processing whereby speech is simplified into word pairings (verb and/or noun combinations) based on adjacency. Given the volume of free response data (approximately 60,000 person-weeks of follow-up) in the LPG and BioLite arms, the bigrams facilitated a systematic approach to exploring the free responses and categorization of the response patterns. We use these qualitatively, to understand potential reasons for non-intervention stove use. Six categories of response emerged from the data: (1) device breakage, (2) food quantity, e.g. cooking for a larger group or having guests; (3) food types, e.g., stirring, frying, or boiling; (4) not home (traveling), (5) speed issues, e.g., being in a hurry, late to work, or stove cooks slowly; and (6) fuel supply, wet or insufficient wood (BioLite) or empty cylinders (LPG). Words that contribute to each category can be found in supplemental table 1.

### 2.4. Stove use monitoring Pre/Post GRAPHS closeout

#### 2.4.1. Spatial data and analysis

Observational research in northern Ethiopia found that distance to forest was a potential confounder in traditional versus improved stove selection, and that adopters appreciated lower solid fuel collection times post intervention (Abadi et al 2017). This maybe because individuals with ready access to solid fuels spend less time on collection, therefore the benefits of an intervention stove (reduction in biomass use and therefore fuel collection) are less valued. We tested this theory using remotely-sensed tree canopy data in 2010 from the Global Forest Change dataset as a proxy for fuelwood availability (Hansen et al 2013) (supplemental figure 3). Each thirty-meter grid cell represents the proportion of tree cover, which is defined as canopy closure for vegetation taller than 5 meters. Geocoordinates for study participants were drawn from the Kintampo Demographic and Health Surveillance System (Owusu-Agyei et al 2012) and were used to create radial spatial buffers from 1 to 4 kilometers. Spatially-weighted averages were calculated within household-specific buffers. Summary statistics for the 3-kilometer buffer averages are available in supplemental table 2. Spearman correlations were then calculated between questionnaire data on self-reported time collecting wood in a week, and the proportion of tree canopy in the buffer. The buffer with the strongest correlation was included as an independent variable in analyses.

#### 2.4.2. Stove use monitoring and data processing

Stove use was tracked with iButton temperature loggers (Model Number DS1921G, Maxim Integrated, San Jose, CA, USA) that recorded temperature in degrees Celsius every ten minutes. Fieldworkers retrieved data every two weeks using Thermodata data downloaders (Thermodata, Eight Mile Plains, QLD, Australia).

Raw temperature data was transformed into a ‘cooking events’ variable using the *AnomalyDetection* package in R. While this package was originally developed to detect anomalies in internet traffic, when applied to temperature data, the package detects events that deviate from the ambient diurnal temperature pattern. We applied numerous filters to the processed data, including only considering positive slope anomalies as cooking time and grouping anomalies within 60 min of each other. Stove use was defined as an indicator variable of 0 (no stove use) or 1 (stove use) in each week. Dates were transformed from calendar dates to weeks relative to each participant’s study end date because GRAPHS had a rolling enrollment and exit.

#### 2.4.3. Time-to-event analysis

We leveraged longitudinal sensor data to perform a time-to-event analysis via Cox proportional hazards regression. A Cox model is used to assess group differences in the time to a binary outcome, considering follow up time and censoring. The measure of effect from this model is a hazard ratio which, if positive, indicates an increased hazard/decrease in the time to the outcome of interest. The outcome of interest is the first week of discontinued intervention stove use, defined as the week after the last recorded intervention cookstove use during the 12 months of stove use monitoring. Univariable regressions were performed for demographic and household-level characteristics with pre-established associations with stove use (Lewis and Pattanayak 2012, Rehfuess et al 2014, Puzzolo et al 2016), including: household wealth (asset index), maternal education, maternal independent income, ethnicity, religion (Christian/non-Christian), number of individuals in household, and fuel collection time. We also included the tree canopy data from our spatial analysis to explore potential ecological drivers of clean/improved cookstove use.

### 2.5. Data integration and analyses

All data analyses were performed in R version 3.5.1. Text analyses were conducted with the *tidytext* package. Spatial analyses were conducted using the *raster* and *sf* packages. Stove use data processing was done with the *AnomalyDetection* package, and time-to-event analyses were performed with the *survival* and survminer packages.

## 3. Results

### 3.1. Description of the cohort and participants

Table 1 outlines participant characteristics of the entire GRAPHS cohort and the sub-cohort of BioLite and LPG users with SUMs data. Sub-cohort participants share similar characteristics to the overall GRAPHS cohort. The most notable differences are between the sub-cohort arms, where BioLite and LPG users have different proportions of ethnic and religious groups represented. Another notable feature of the entire GRAPHS cohort is that the female participants had more years of education than their male partners. Correlations between continuous variables can be found in supplemental figure 2.

### 3.2. Self-reported stove use during GRAPHS

#### 3.2.1. Self-reported intervention stove use

**Figure 3.**
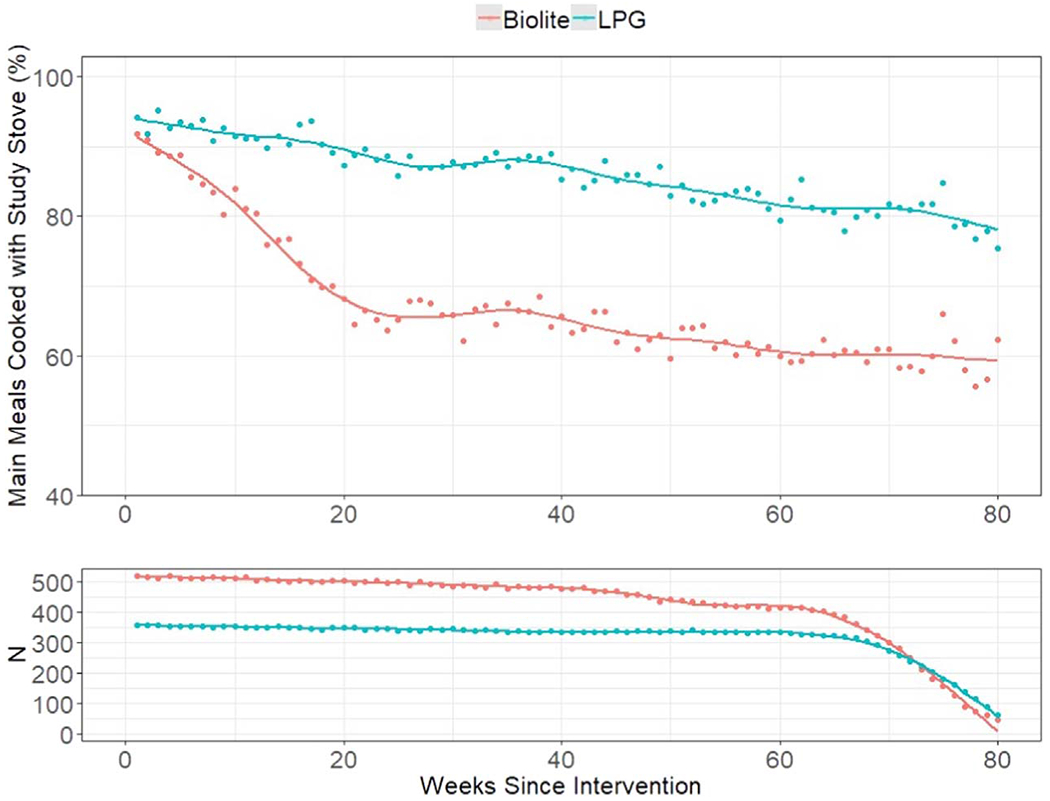
Weekly self-reported intervention stove use for main meals throughout GRAPHS. Top = proportion of main meals reported as cooked with intervention stove. Bottom = Number of households responding. Points are observations and the lines are locally weighted regression smoothers.

In total, 59,344 participant-weeks of survey data were collected. There were marked differences in self-reported stove use patterns during GRAPHS across study arms (figure 3). There was near universal stove compliance for both arms at the beginning of GRAPHS. However, over the course of participant enrollment in the study, self-reported stove use decreased for each study arm, but more substantially for the BioLite. Intervention stove use was reported in 87% of participant-weeks among the LPG study arm and 69% of participant-weeks for the BioLite, with higher use during pregnancy than post-pregnancy (supplemental table 3). By the end of GRAPHS, 60% of BioLite study arm participants reported using their stove, whereas at the end of the study, 80% of LPG arm participants report intervention stove use. Disenrollment from the study was staggered between 60–80 weeks.

#### 3.2.2. Self-reported reasons for traditional stove use

**Figure 4.**
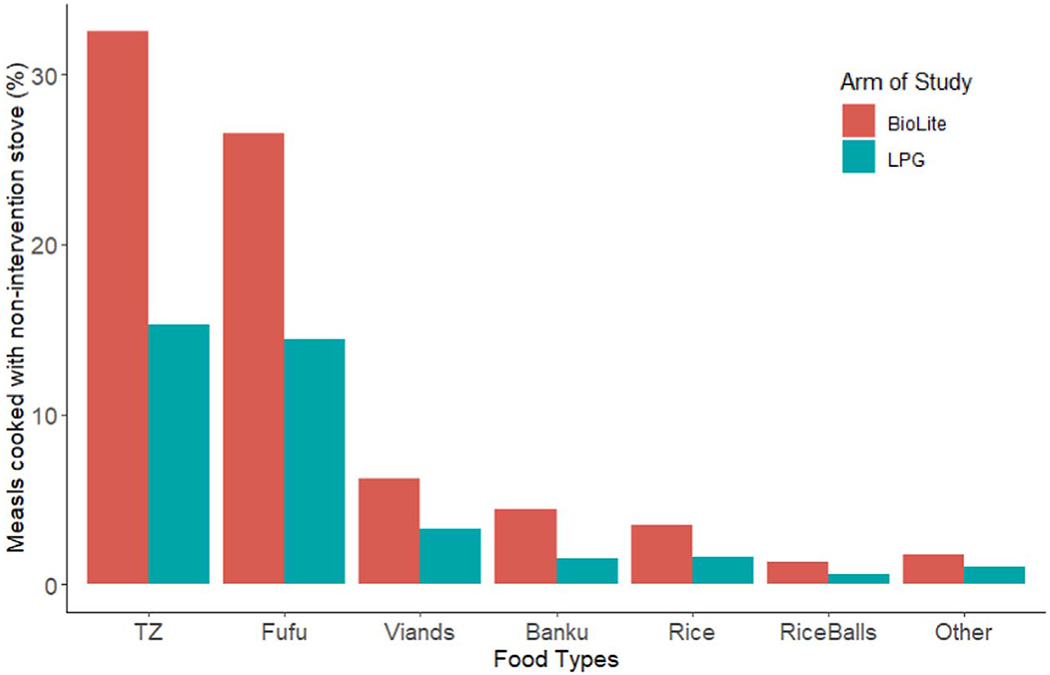
Foods cooked with non-intervention stoves. *TZ = Tuo Zaafi, Fufu and Banku = thick starches often eaten with soups and stews, Viands = boiled starchy vegetables (cocoyam, plantains, cassava,* etc). Values do not sum to 100% due to participants who chose not to respond.

Participants who reported use of a traditional non-intervention stove (3-stone fire or charcoal) for some meals were asked which foods were cooked during these meals. Participants reported cooking tuo zaafi (TZ) and fufu with non-intervention stoves in higher proportion than other food types in both study arms, though this pattern was more pronounced for BioLite rather than LPG users (figure 4). These foods both require time-intensive preparation and consist of a pounded and thickened starch, served alongside a soup or stew.

Each week, participants were also asked whether they had sustained any burns while cooking with the intervention stoves. Five percent of individuals experienced burns in the LPG arm (n = 20), compared to 20% BioLite participants (n = 101), and 9% control arm participants (n = 45). Burns were associated with decreased intervention stove use for BioLite (RR: 0.958, p = 0.009) in an analysis of self-reported stove use stratified by burn experience (table 2). This pattern was not observed among LPG users (p = 0.975).

#### 3.2.3. Text analysis

**Figure 5.**
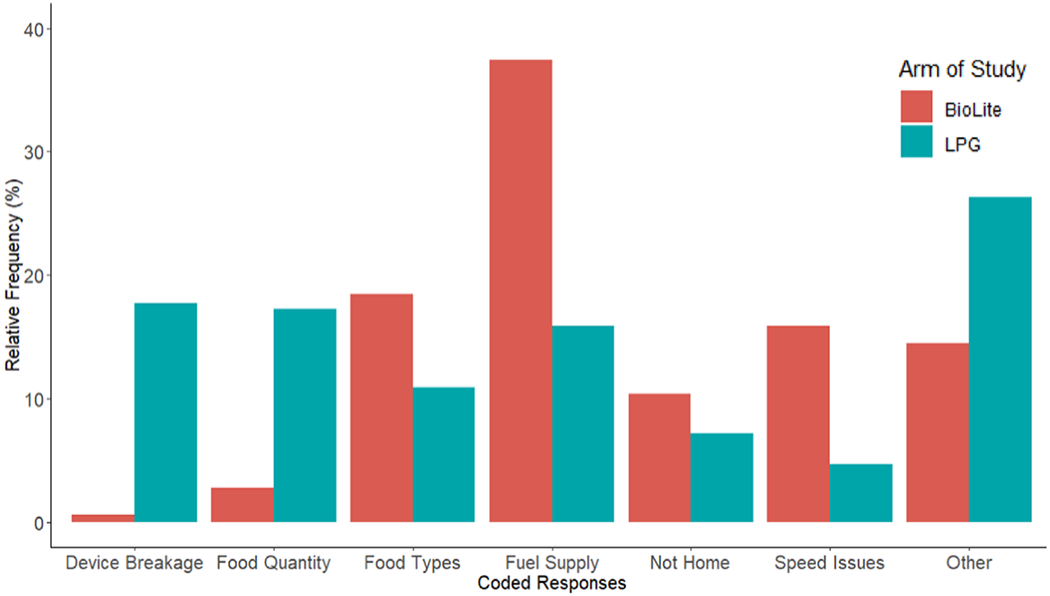
Results of text analysis from open-response question regarding reasons for not using intervention stoves in the past week. Synthesized from bigrams depicted in supplemental figures 4 and 5. Words categorizations in supplemental table 1.

Text analysis of reasons for non-intervention stove use yielded different patterns for LPG (supplemental figure 4) and BioLite (supplemental figure 5). Bigrams were grouped into six themes (figure 5).

- **Device breakage** was mentioned in 18% of bigrams from LPG users, but only in 1% of BioLite bigrams. For LPG users, stove breakages included faulty regulators, leaking gas, and broken tubes.
- **Food quantity** was also mentioned frequently, appearing in 17% of LPG and 3% of BioLite bigrams. Individuals may have been cooking for more than their immediate family, including social gatherings like funerals or for farm laborers.
- **Food types** refers to specific meals or preparation styles that were found to be reasons for non-intervention stove use. We found that participants perceived preparation styles like frying and stirring as challenging to carry out with intervention stoves.
- **Fuel supply** refers to access challenges for both intervention stove fuels. In the case of BioLite, almost 40% of bigrams mention a fuel supply issue, including fuelwood shortage, wet firewood, or not having wood pieces that were small enough for the intervention stove.
- **Not home** refers to travel or sleeping elsewhere as a reason for non-intervention stove use. For example, study participants report sometimes sleeping at farm plots that are remote from their primary home and intervention stove.
- **Speed Issues** refers to the fact that many women reported getting home late, leaving the home early, or otherwise being in a hurry as reasons not to cook with the stove. We referred to these reports as speed issues and found that 16% of BioLite and 5% of LPG bigrams mention such challenges.

### 3.3. Stove use monitoring Pre/Post study closeout

#### 3.3.1. Stove use patterns

**Figure 6.**
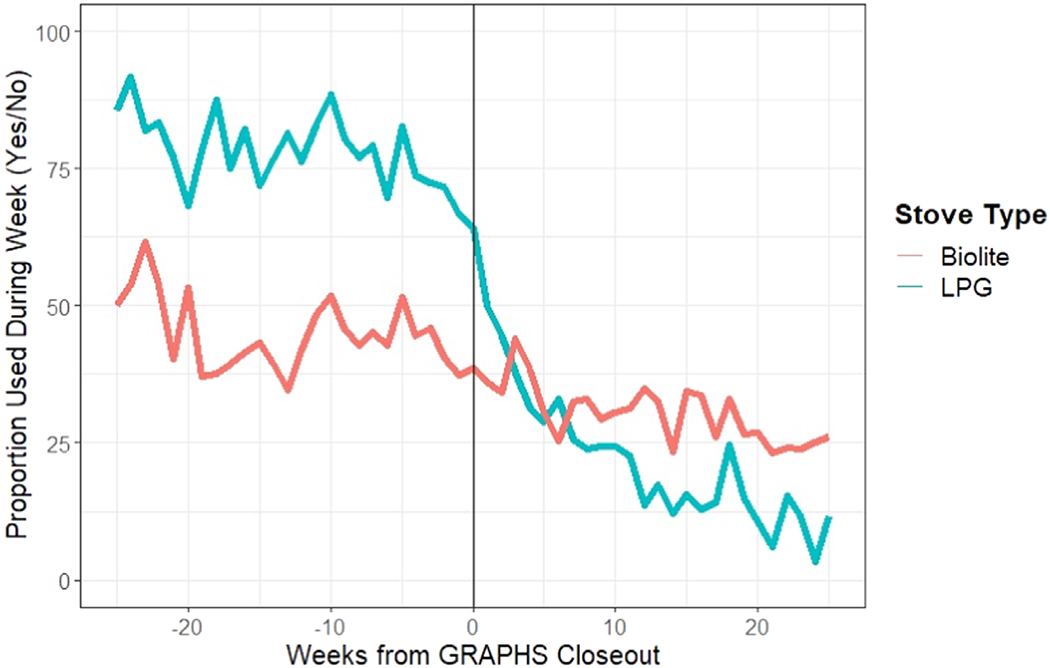
Proportion of measured stove use (from stove use monitors) in a given week relative to the GRAPHS study end date.

Participants’ stove use was tracked via SUMs in the final 6 months of GRAPHS and 6 months after study closeout to understand patterns of discontinued intervention stove use (figure 6). Since the LPG arm was provided with free refills during the study period, but not after, we hypothesized that LPG use would decrease significantly after study termination. However, we found that LPG use seemed to decline before the study termination date. BioLite use was consistently lower than LPG before GRAPHS closeout. After GRAPHS closeout, BioLiteuse was consistently higher than LPG use.

**Figure 7.**
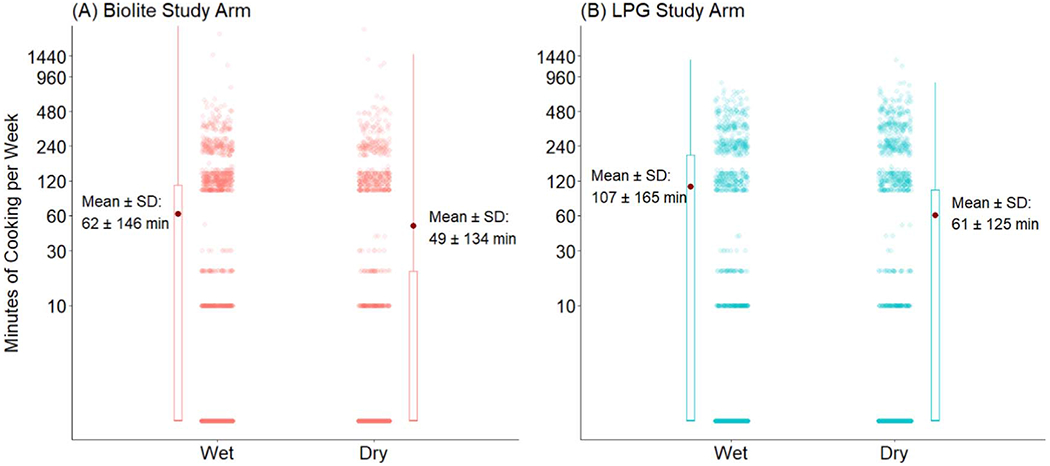
Minutes of stove use per week in the subcohort by stove and season (*y* axis is spaced logarithmically). Dark red point = mean, dots = observations of minutes cooking per week. Box = interquartile range (IQR), whiskers = 1.5 times the IQR. Median of each group = 0 min.

Stove use also varied seasonally (figure 7). BioLite users appeared to use their stoves more during the wet season (mean = 62 min/week) compared to the dry season (mean = 49 min/week). This trend was more pronounced for LPG participants, who used their stoves more during the wet season (107 min/week) than the dry season (61 min/week). Notably, the median was 0 min/week for both arms for both seasons. There was also evidence of a bi-modal distribution of stove use (supplemental figure 6). Both stoves have a lower mode of 10 min per week, which is the lowest detectable stove use given the device sampling frequency. The higher mode for BioLite was 120 min per week and 230 min for LPG. There is also a mode at zero, implying those who did not use their stoves on given days or had already discontinued stove use.

#### 3.3.2. Time-to-discontinuance analyses with cox regressions

**Figure 8.**
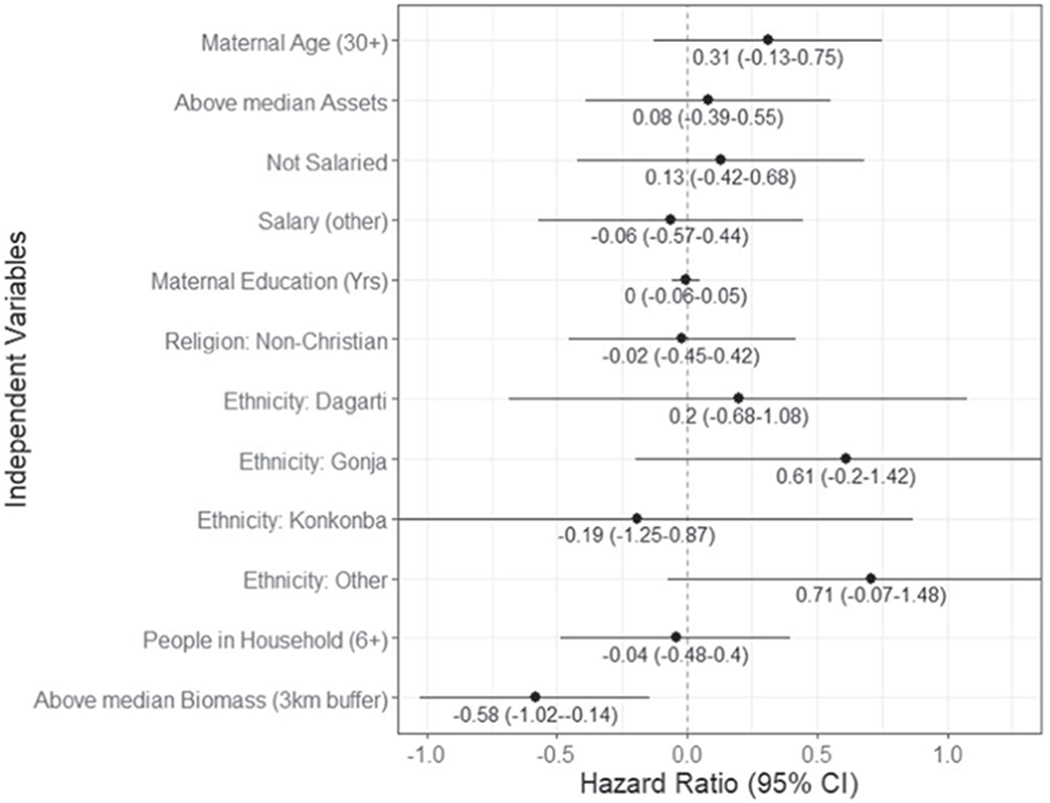
Univariable Cox proportional hazard regression coefficients: Hazard ratios with 95% confidence intervals. Outcome is stove use discontinuance (week after last measured use). Positive values indicate increased discontinuance by the end of the study period, negative indicate decreased discontinuance. n = 103.

We conducted univariable Cox proportional hazard regressions among LPG users to understand the relationships between household, demographic, and ecological variables and discontinued clean stove use for the LPG sub cohort (figure 8). We focus on LPG due to its high use during GRAPHS, relevance to current Ghanaian policy, and the removal of the GRAPHS fuel subsidy. Univariable results for the BioLite stove can be found in supplemental figure 8.

**Figure 9.**
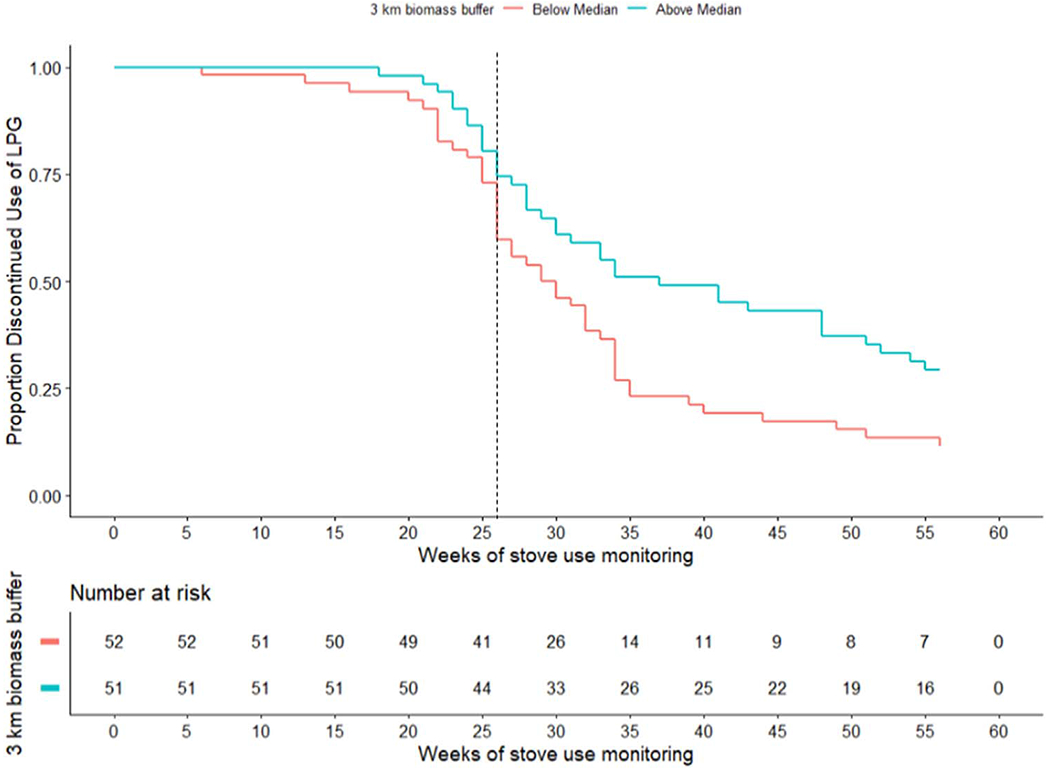
Time-to-event curve comparing discontinued use of LPG for those above the median tree canopy in a 3-kilometer buffer, and below. Dashed line at the end of GRAPHS (no additional LPG refills paid by study).

The time-to-event analysis starts at 6 months prior to the end of each participant’s enrollment in GRAPHS and continues for 6 months following the GRAPHS study end (supplemental figure 9). Most covariates showed no meaningful association with stove use discontinuance. However, individuals above the median proportion of tree canopy (19%) in a 3-kilometer buffer were less likely to discontinue use of the LPG stove by the end of the study compared to those with below median tree canopy (HR_*unadjusted*_ = −0.58, p < 0.001). We focus on a 3-kilometer buffer because this radius has the strongest relationship with self-reported fuelwood collection time in the full GRAPHS cohort (supplemental table 4). Statistical significance persisted and the magnitude strengthened when adjusted for potential confounders like the asset index, household size (number of householders), and participant’s occupation (farmer versus not) (HR_*adjusted*_ = −0.646, p = 0.007). The median time-to-discontinuance for those above the median tree canopy was 37 weeks while those below was 29.5 weeks (figure 9).

## 4. Discussion

**Figure 10.**
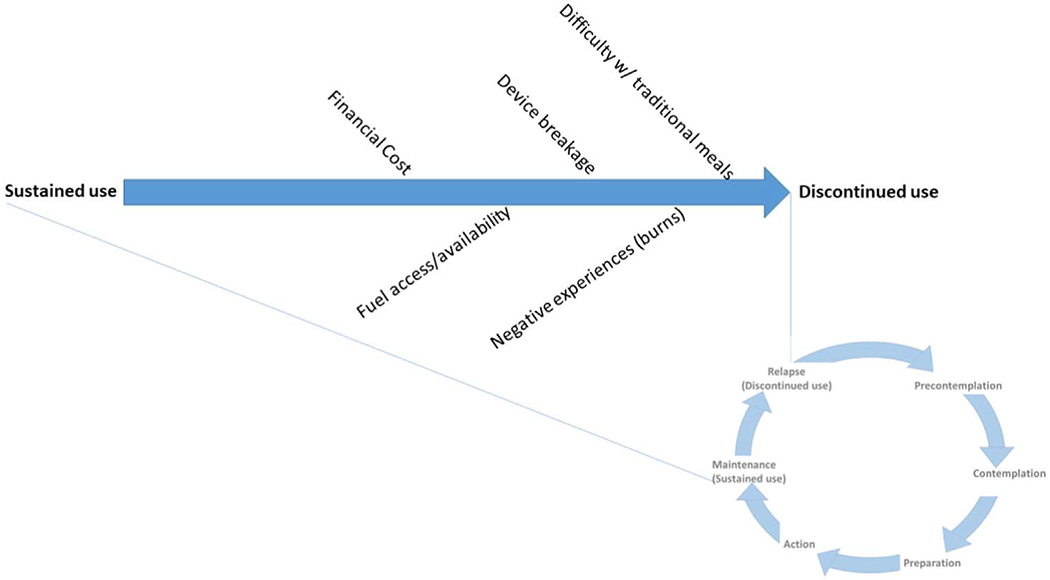
Summarized findings: impediments to sustained use and potential reasons for discontinuance.

We conducted a study in a rural area of Ghana with robust longitudinal data from surveys and sensors to analyze patterns of intervention stove use by study arm, including barriers to use, and the factors related to discontinued clean cookstove use. Survey responses during the study period allowed us to characterize difficulties encountered when LPG refills were free and fieldworkers maintained/ repaired LPG stoves when necessary. Cox time-to-event analyses allow us to identify the factors that inform clean cookstove discontinuance when these supports were removed. We summarize our findings in the conceptual diagram (figure 10).

Overall, use of intervention stoves was relatively high during GRAPHS, especially for LPG, though use decreased after pregnancy. Our research supports findings from past studies that stoves maybe perceived as suitable for some cooking tasks, but not others (Grimsby et al 2016, Piedrahita et al 2016, Hollada et al 2017, Gould and Urpelainen 2018, Dickinson et al 2019). The design of future interventions should respond to specific, culturally valuable foods. In this case, traditional Ghanaian cuisine includes thick starchy foods that accompany soups, such as banku, fufu, and tuo zaafi. These dishes, often cooked in heavy pots, require constant stirring over a fire. 3-stone fires may provide a more stable base for stirring thick foods, and their fires could be more easily sustained with larger fuelwood for time-intensive meals. It is also possible that individuals prioritize intervention stoves for faster, lower-intensity dishes, in order to maximize limited LPG supply. Alternatively, participants may not know how to prepare these meals with BioLite or LPG stoves. Given the required stirring, this could result in mistakes in preparation and handling, perhaps resulting in accidental burns. We found that only 5.5% of LPG users experienced burns, a lower proportion than on traditional 3-stone fires (9%). However, 20% of BioLite users reported burns. Although improved and clean cookstoves are seen as a means of reducing burns (Simon et al 2014), a burn from an intervention stove may still deter future use.

We also demonstrate that text analysis can effectively characterize open-ended responses on motivations of stove use patterns not otherwise captured by structured survey questions. We found that the most common reasons for use of non-intervention stoves among LPG participants were device breakage and food quantity. Device breakage concerns could be partially reflective of fears regarding the safety of LPG, which researchers have observed in other parts of the world (Budya and Yasir Arofat 2011, Kimemia and Annegarn 2016). BioLite users mostly discussed fuel supply concerns. For example, many BioLite users report fuel supply issues due to wet wood. It is possible this refers to green, rather than dead/dried, firewood. Past literature has found that most Ghanaians in this region use green firewood due to scarce wood supply (Amoah et al 2019). The BioLite stove requires small, dry pieces of firewood for efficient combustion. Additionally, even dry firewood could be rained on during the wet season. We found clear seasonal stove use differences for LPG and BioLite, with higher use in the wet season. Seasonal stove use patterns have been reported elsewhere (Lam et al 2017). LPG and BioLite stoves are more mobile than 3-stone fires, so individuals may appreciate the ability to bring them indoors during the rains. It is also possible that participants can afford to refill their stoves during the harvest season, rather than during the financially leaner dry season. The average monthly household income in rural savannah areas of Ghana is 841 GH£ (Ghana Statistical Service 2014) and the cost to refill a 16 kg cylinder was 69 GH£, or 8% the monthly average income (Carrión et al 2020).

Time-to-event analyses showed that the only variable associated with intervention stove use discontinuance was the proportion of tree canopy in a 3-kilometer radius of the household. This is interesting, given that we explored many variables that have been associated with sustained use in past studies, including wealth, education, income, ethnicity, religion, and household size (Puzzolo et al 2016). Our analysis demonstrates that high biomass availability is associated with sustained LPG use, which was the opposite of our hypothesis. It is possible that the loss of free LPG refills increased stove stacking (concurrently use traditional stove types) to ration LPG, which would be more likely to happen when firewood is more easily accessible. This theory is supported by high overall use during the time period where LPG was free to participants; and recent qualitative evidence shows that individuals in this cohort like their LPG stoves (Agbokey et al 2019).

### 4.1. Limitations

There are limitations to our analysis worth considering. First, we are unable to assess stove stacking with these data. For the health benefits of clean cookstove adoption, it is imperative that we decrease both stove stacking *and* discontinuance of clean cookstoves. While we had intended to collect data on traditional stove use, 3-stone fires presented challenges leading to substantial device breakage and data loss. Second, while our remotely-sensed dataset offers a fine-resolution estimate of tree canopy, it may not completely address the underlying data need. While it is possible that individuals travel into dense bush to collect wood, it is likely that they also travel to the closest living trees for green firewood (Amoah et al 2019). Such trees are likely at the forest edge, which is not captured in the remotely-sensed data. Furthermore, radial distance to biomass is a simple construct, and past studies have shown that more complicated dynamics are likely (Brouwer et al 1997, Köhlin et al 2001, Singh et al 2018). Longitudinal firewood consumption patterns in this region would be instructive (Amoah et al 2019). A third limitation is our lack of survey data in the post-GRAPHS time period. One particular area of interest is perceptions of health risk as a driver of sustained use. Although we did not address this in this study, we have forthcoming research to address this topic (Carrión et al 2018). Fourth, we do not explicitly capture the role of fuel price on discontinued use. Instead we are observing use during the LPG subsidy compared to afterward. Other elements of our intervention could influence use, including weekly fieldworker visits. This, combined with our lack of post-GRAPHS survey data, means that we do not know if any participants refilled their cylinders. Fourth, the tree canopy dataset is derived from the Landsat 7 satellite which has a documented error resulting in as much as 20% data loss in a retrieval. Hansen *et al* conducted a gap-filling approach for these missing values, which we believe would ultimately cause nondifferential measurement error, and thus should not bias our estimates. Finally, we have a measure of wealth, but not income. Income changes overtime, especially in an agricultural setting where income may be seasonal, and those fluctuations could be instructive in understanding reasons for discontinued cookstove use. Future studies of clean cookstove discontinuance should consider ways to measure income over time.

## 5. Conclusion

To our knowledge, this is the first study to utilize the stove use discontinuance framework, which has its foundations in health behavior theory. We utilized longitudinal survey and sensor data to understand the discontinuance of clean cookstoves in a rural Ghanaian cohort. Our findings suggest that device breakage, food types, and fuel costs—including access and availability—influence intervention stove disuse and discontinuance. Additional efforts should be made to understand the role of biomass availability on discontinued stove use, which is particularly germane in a region with deforestation concerns. Evidence suggests LPG is widely accepted, but the recurring cost of fuel is likely prohibitive. Given that Ghana, and many other countries, are trying to scale up the use of clean cooking fuels, we recommend that future studies employ the clean cookstove discontinuance framework to appreciate the full ecological and health benefits of clean cooking transitions.

## Supplementary Material

Supplemental information

## Figures and Tables

**Table 1.** Demographic and household characteristics of the GRAPHS cohort and the sub-cohort (BioLite and LPG) tracked with stove use monitors (SUMs). Continuous variables reported as mean (standard deviation) and categorical variables as count (percentage).

	GRAPHS Cohort	BioLite	LPG
n	1412	117	103
Household size (number Of householders)	6.54 (3.57)	6.31 (3.1)	5.90 (2.28)
Ethnicity			
Akan	243 (17.2)	22 (18.8)	11 (10.7)
Dagarti	314 (22.2)	19 (16.2)	18 (17.5)
Gonja	217 (15.4)	13 (11.1)	26 (25.2)
Konkonba	192 (13.6)	21 (17.9)	10 (9.7)
Other	446 (31.6)	42 (35.9)	38 (36.9)
Religion			
Christian	864 (61.2)	74 (63.2)	57 (55.3)
Muslim	421 (29.8)	27 (23.1)	41 (39.8)
Other	127 (9.0)	16 (13.7)	5 (4.9)
Marital Status			
Married	777 (55.0)	74 (63.2)	61 (59.2)
Living together, unmarried	458 (32.4)	31 (26.5)	29 (28.2)
Single	177 (12.5)	12 (10.3)	13 (12.6)
Participant’s (Female) Years of Education	6.65 (5.57)	7.28 (5.46)	6.54 (5.63)
Husband/Partner (Male) years of Education	1.68 (1.98)	1.91 (2.01)	1.79 (2.03)
Household Asset Index	0.00 (1.95)	−0.23 (1.61)	−0.45 (1.41)
Participant’s Age	29.01 (7.17)	30.11 (6.72)	29.03 (6.76)
Hours per week Collecting Wood	6.41 (6.43)	5.77 (5.91)	5.02 (5.58)

**Table 2.** Stove use stratified by individuals who report burns from the intervention stove compared to those who report no burns. Rate ratios and p values calculated from quasi-poisson regression with per person days of use as the dependent variable. Bold results have a p-value < 0.05.

Fuel Type	Burns	Weeks used	Total weeks	Proportion use	Risk ratio	P value
LPG	No Burns	20232	23298	86.8%	Reference	0.975
	Burned	1204	1461	82.4%	1.001	
BioLite	No Burns	18621	26945	69.1%	Reference	**0.009**
	Burned	4389	6590	66.6%	**0.958**	
