# Supplementary material for: Using longitudinal survey and sensor data to understand the social and ecological determinants of clean fuels use and discontinuance in rural Ghana
Source: Environ Res Commun. Author manuscript; Available in PMC 2021 Sep 8. (PMC8425314; doi:10.1088/2515-7620/abb831)

Supplemental Materials

Supplemental Figure 1: Weekly stove compliance survey questions administered for all participants during the Ghana Randomized Air Pollution and Health Study (GRAPHS).

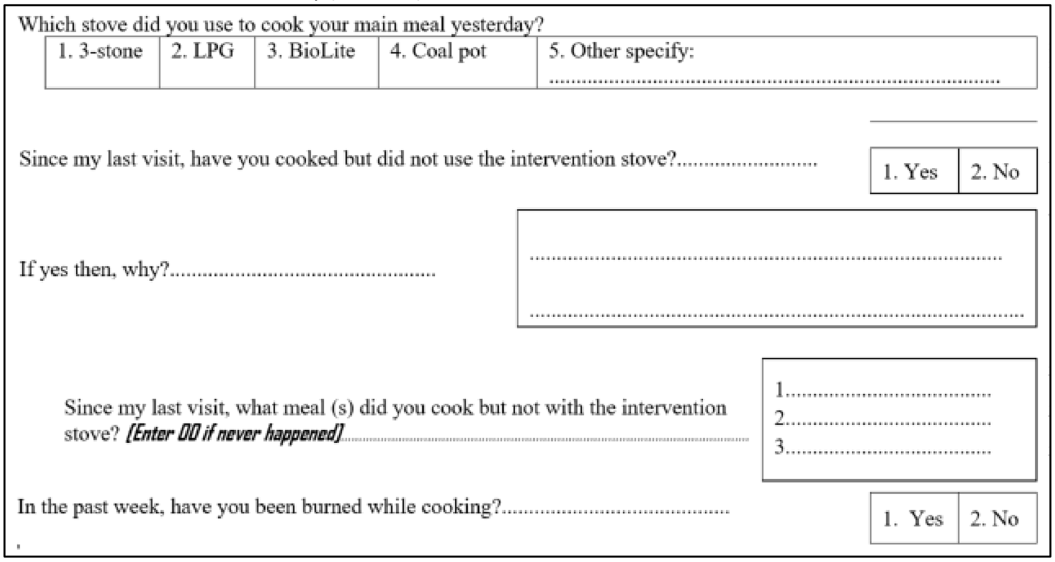

Which stove did you use to cook your main meal yesterday?

1. 3-stone	2. LPG	3. BioLite	4. Coal pot	5. Other specify: ....

Since my last visit, have you cooked but did not use the intervention stove?.......................... 1. Yes | 2. No

If yes then, why?... ....

Since my last visit, what meal (s) did you cook but not with the intervention stove? *[Enter 00 if never happened]*........ 1.................. 2.................. 3..................

In the past week, have you been burned while cooking?... 1. Yes | 2. No

Supplemental Table 1: Words that contribute to bigram analysis categorization.

Category	Stove Type	Words
Device breakage	LPG & BioLite	Spoil, spoiled, spoilt, broken, broke, damaged, damage, regulator, leak, leaked, faulty
Food type	LPG & BioLite	TZ, banku, fufu, porridge, coco, koko, fry, boil, boiled, boiling, kenkey, stir, stirring, yam, frying, smoking, smoke, bofrot, pito, beans
Food quantity	LPG & BioLite	People, plenty, visitors, quantity, large, commercial, sell, size, sale, guest, guests
Fuel Supply	LPG	Shortage, fetch, empty, cylinder, spent, refill, refilling, finish, finished
	BioLite	Shortage, wet, dry, fetch, firewood
Not Home	LPG & BioLite	Away, farm, travel, traveling, travelled, left, leaving
Speed issues	LPG & BioLite	Slow, fast, hurry, late

Supplemental Figure 2: Spearman correlation plot for GRAPHS Cohort for variables thought to be associated with clean cookstove and improved cookstove sustained use (N = 1412). *Abbreviations: BiomassBuffer = proportion of tree canopy in a 1 km buffer; HHSize = # of individuals living in the home; FemaleEdu = woman's education (years); MaleEdu = man's education (years); WoodCollect = self reported time collecting wood in a week.*

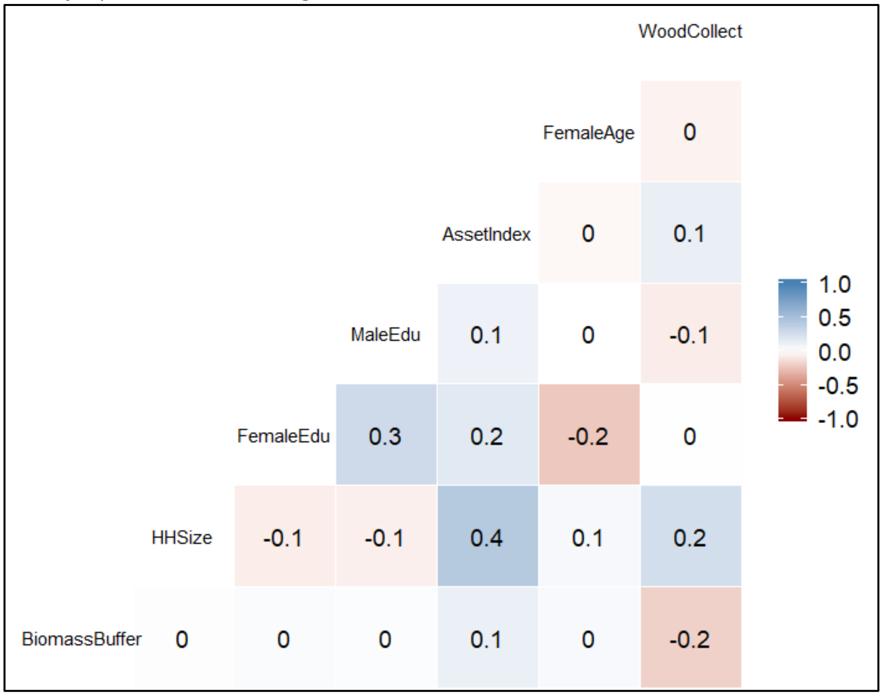

Supplemental Figure 3: Tree canopy in the study region in 2010 with administrative boundaries overlaid.

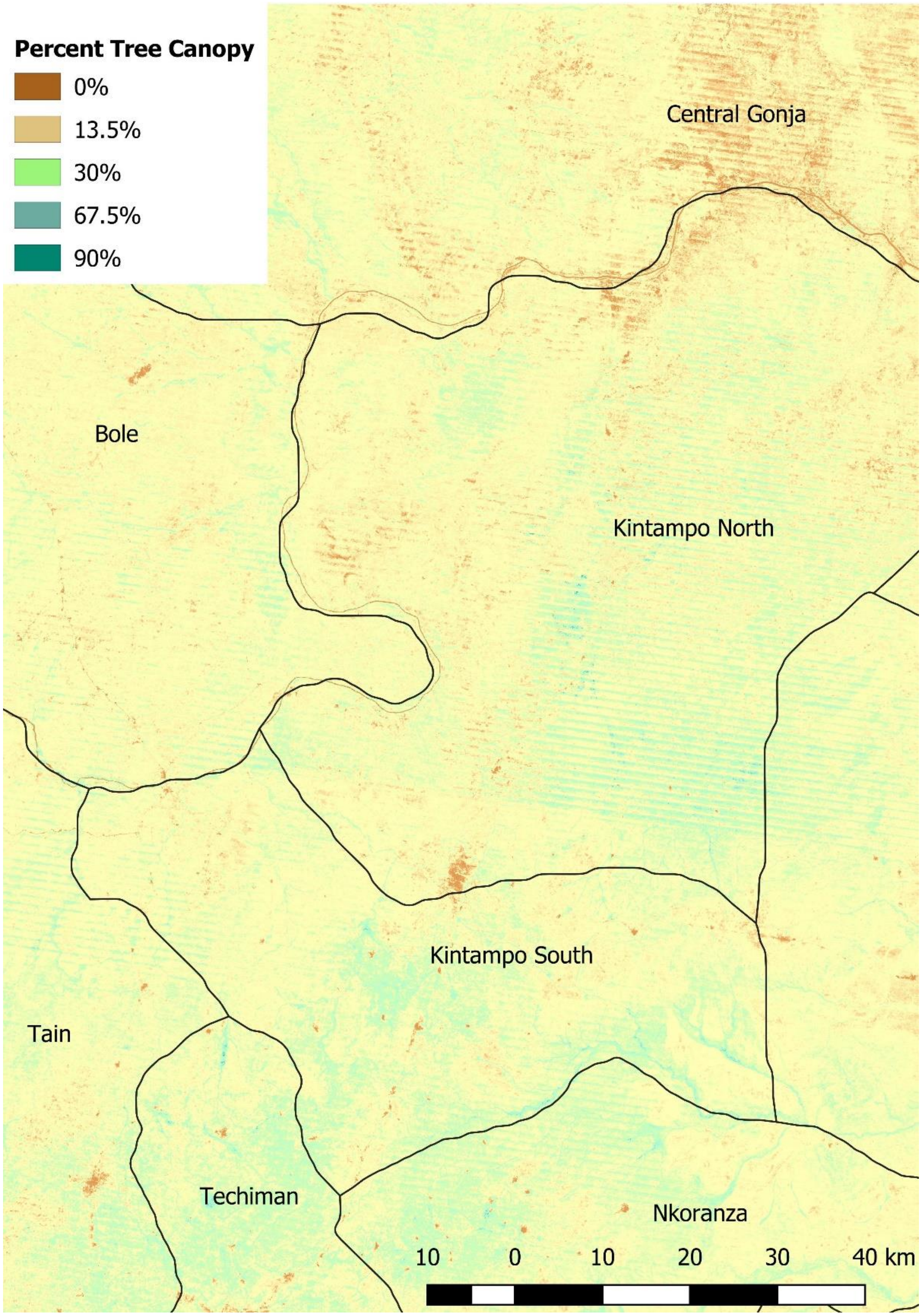

Source: Hansen/UMD/Google/USGS/NASA

Supplemental Table 2: Summary statistics on 3-kilometer buffer tree canopy in cohort.

Group (n)	Mean (std. dev.)	Median	IQR	Range
GRAPHS Cohort (1412)	20.58% (4.92)	19.58%	18.2-22.8%	10.8-31.4%
Biolite (526)	21.2% (5.55)	19.6%	18.9-23.9%	11.5-31.4%
LPG (361)	19.4% (5.53)	19.2%	18.1-21.2%	10.8-31.4%
3 stone (525)	20.8% (3.46)	20.2%	18.2-22.7%	16.1-29%

Supplemental Table 3: Proportion of self-reported intervention stove use by pregnancy status.

Arm of Study (n)	Pregnancy Status	Weeks of Intervention Stove Use	Total Weeks	Proportion Used	
**LPG** (361)	Pregnant	7258	7610	95.4%	86.6%
	Not Pregnant	14689	17735	82.8%	
**BioLite** (526)	Pregnant	8543	9855	86.7%	68.5%
	Not Pregnant	14759	24144	61.1%	

Supplemental Figure 4: Bigrams produced from BioLite participant open responses for reasons why they used a non-intervention stove during the past week. Identified in 5 or more responses. Arrows point from words that are more likely to be the first word in a bigram.

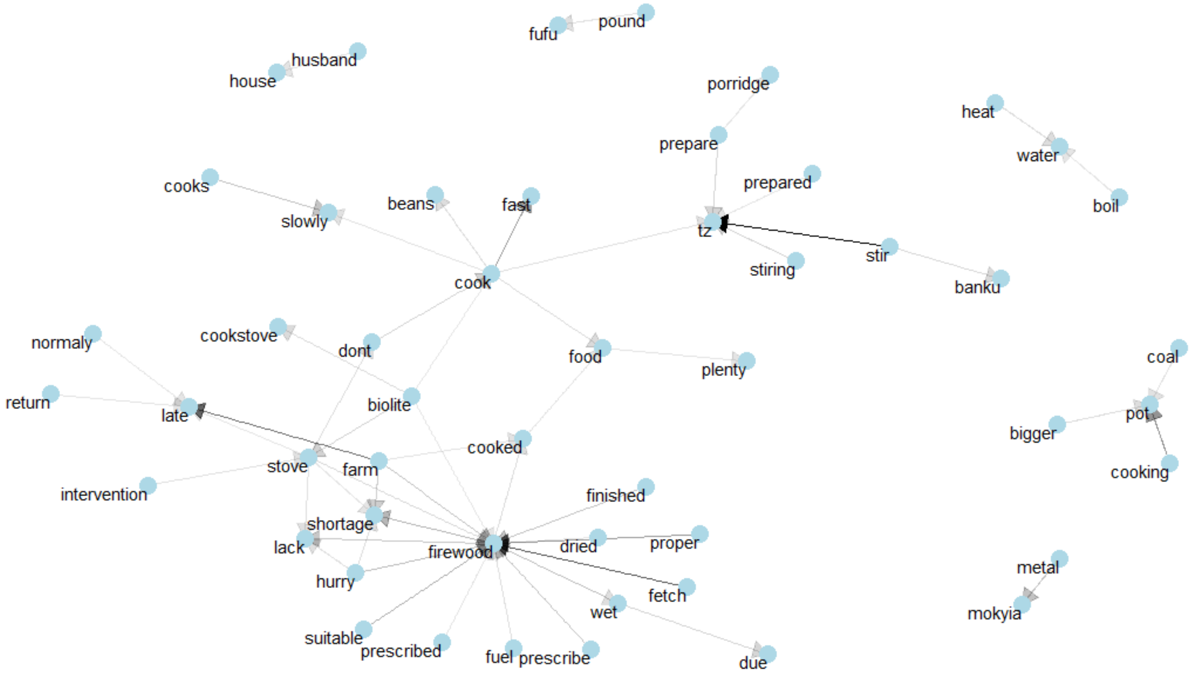

Supplemental Figure 5: Bigrams produced from LPG participant open responses for reasons why they used a non-intervention stove during the past week. Identified in 5 or more responses. Arrows point from words that are more likely to be the first word in a bigram.

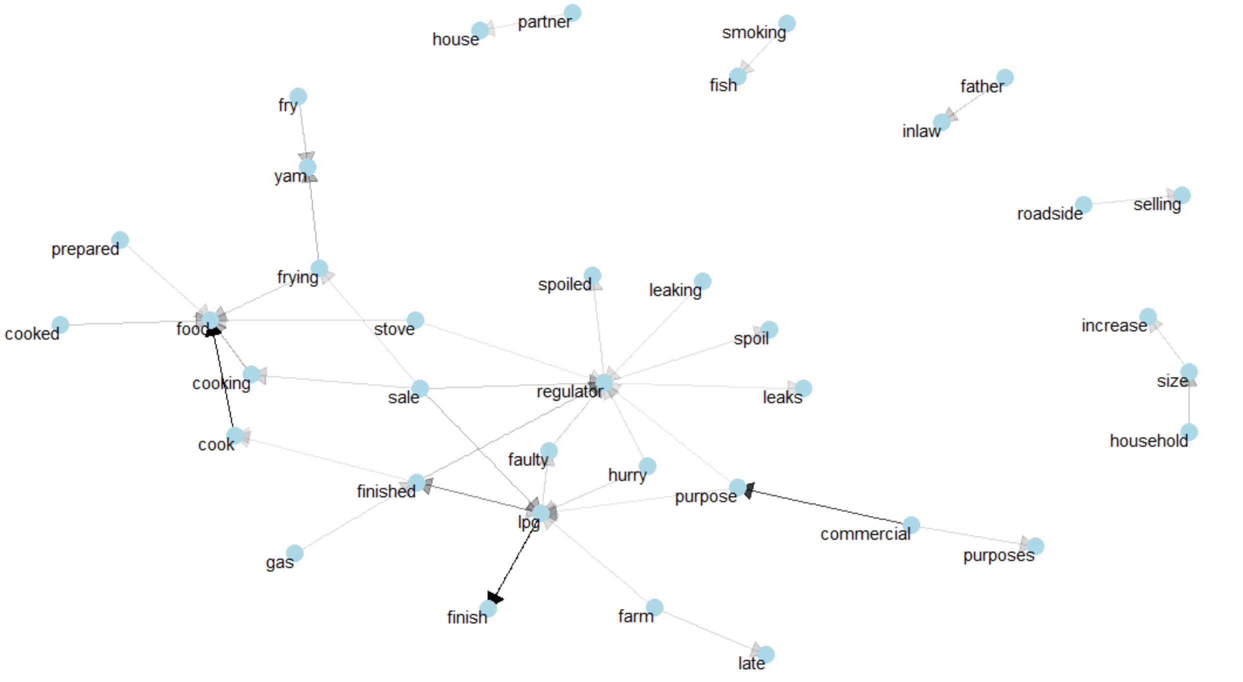

Supplemental Table 4: Correlations for weekly self-reported firewood collection time and proportion of tree canopy within a given distance of the participant's home (N = 1412)

Buffer Size	Spearman's rho	p-value
1 km	-0.170	<0.001
2 km	-0.185	<0.001
3 km	-0.191	<0.001
4 km	-0.184	<0.001

Supplemental Figure 6: Density plot of stove use monitor data (GRAPHS subcohort) from BioLite (n=117) and LPG (n=103) participants.

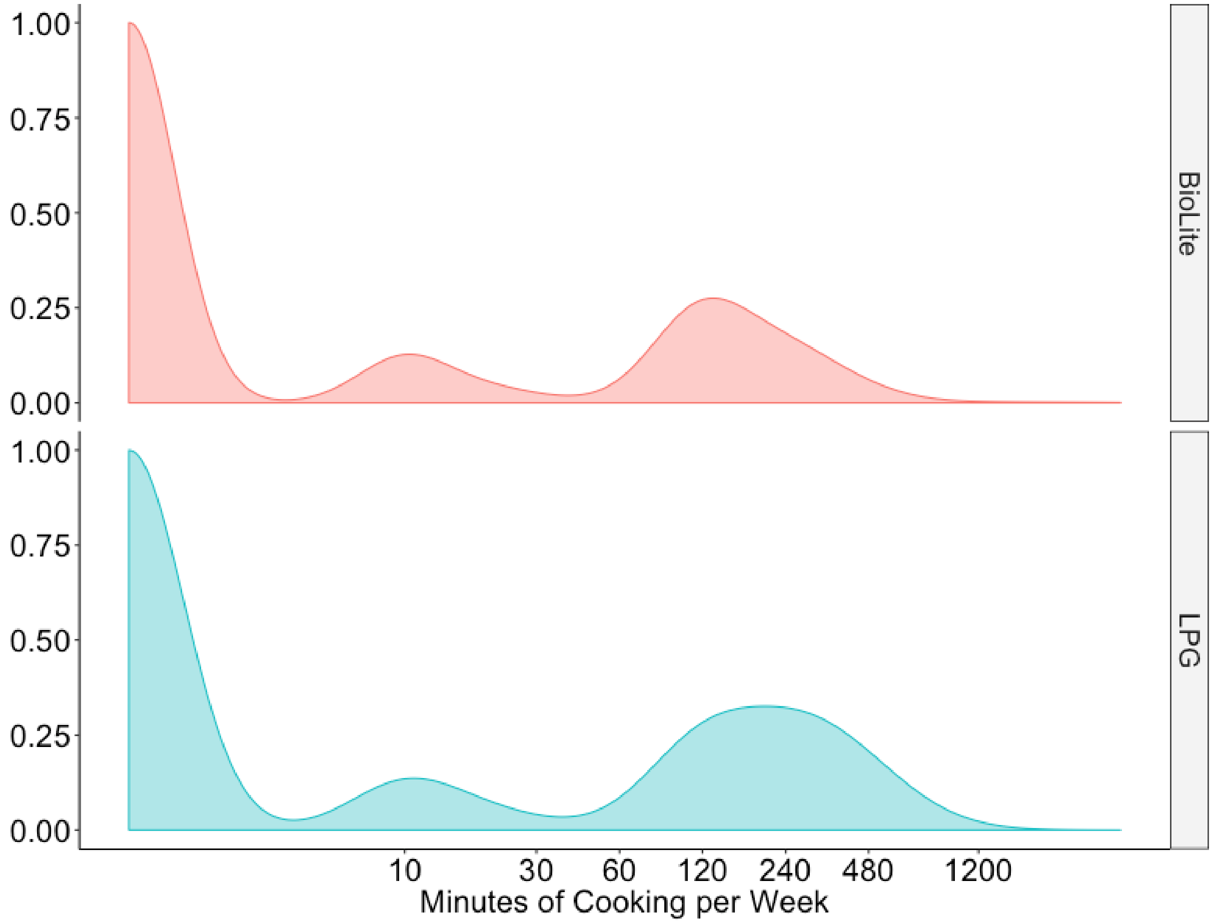

Supplemental Figure 7: Stove use monitoring data for BioLite (n = 117) and LPG (n = 103) participants. The x-axis represents the time to and from the GRAPHS end date. The dots are household weekly observations, and the smoothed line is a local regression curve.

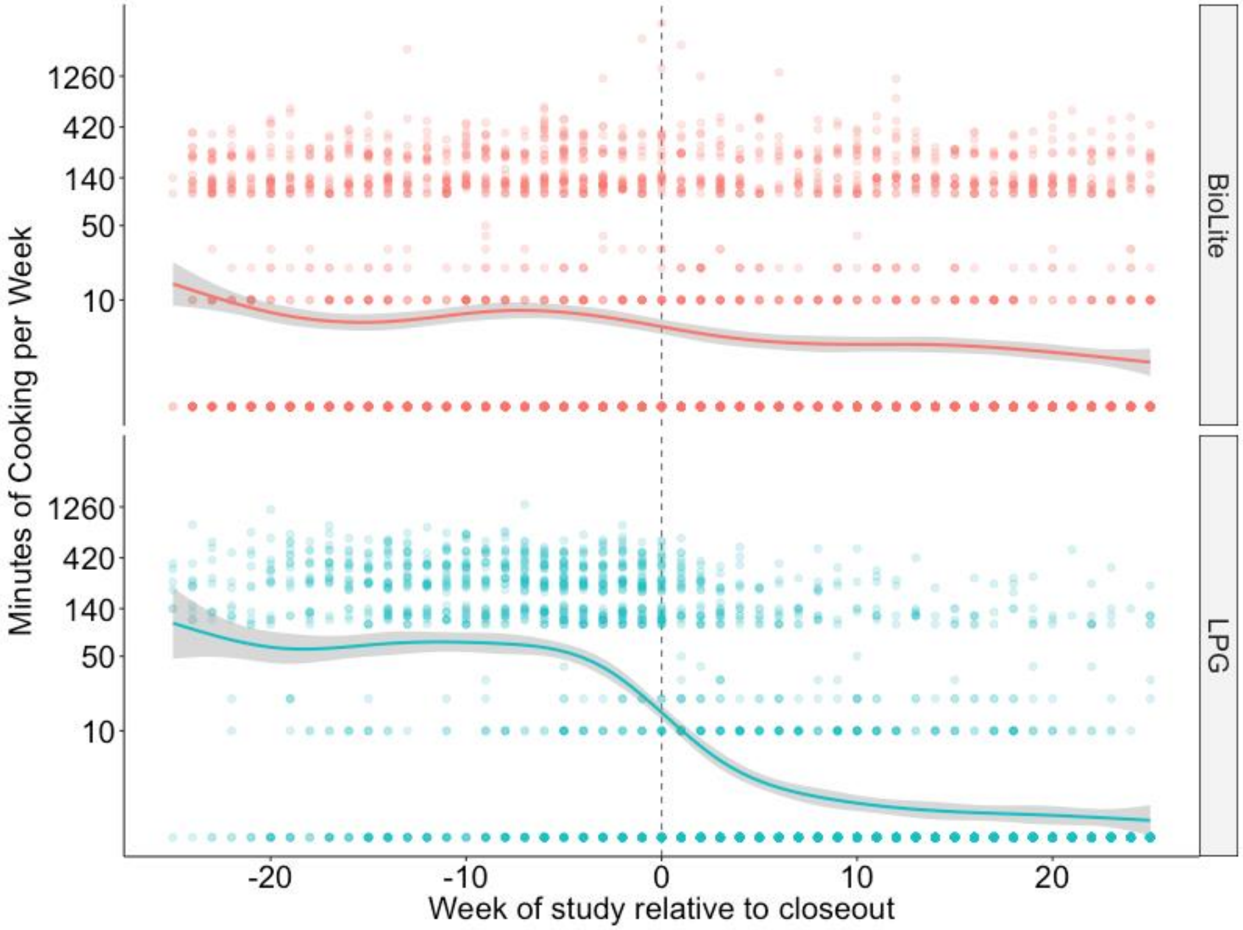

Supplemental Figures 8: Univariable Cox proportional hazard regression coefficients for BioLite users: Hazard ratios with 95% confidence intervals. Outcome is stove use discontinuance (week after last measured use). Positive values indicate increased discontinuance by the end of the study period, negative indicate decreased discontinuance. n = 117.

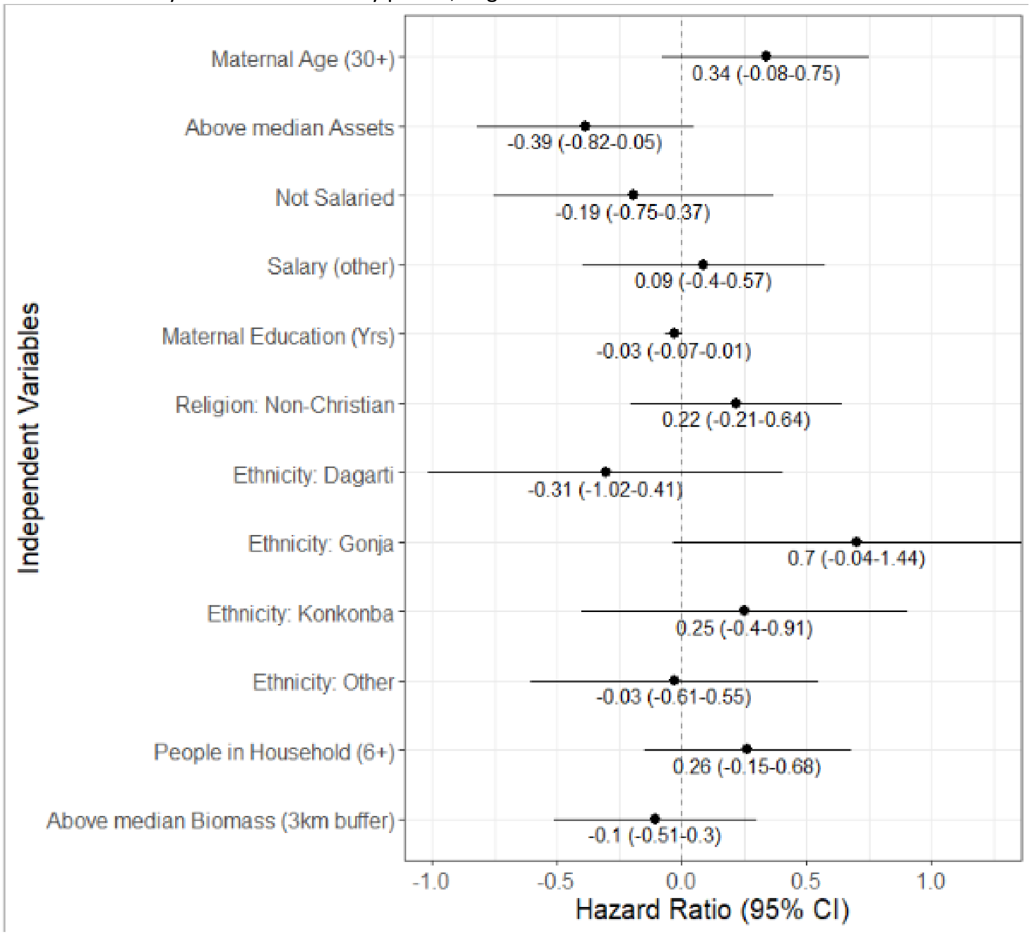

Supplemental Figures 9: Unadjusted time-to-event curve for LPG users (n = 103). The starting point represents 6 months prior to GRAPHS closeout.

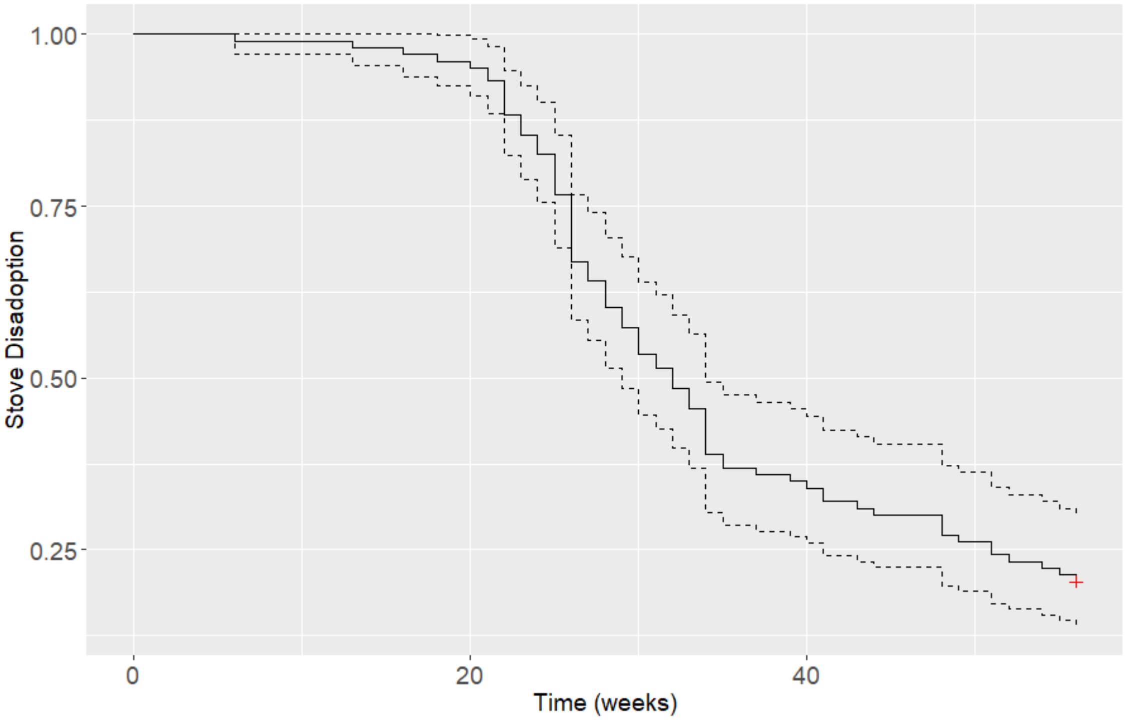

Supplement: Supplemental information [file NIHMS1730613-supplement-Supplemental_information.pdf]
